# Self‐Induced Solutal Marangoni Flows Realize Coffee‐Ring‐Less Quantum Dot Microarrays with Extensive Geometric Tunability and Scalability

**DOI:** 10.1002/advs.202104519

**Published:** 2022-02-07

**Authors:** Jeongsu Pyeon, Kyeong Min Song, Yeon Sik Jung, Hyoungsoo Kim

**Affiliations:** ^1^ Department of Mechanical Engineering Korea Advanced Institute of Science and Technology Daejeon 34141 Republic of Korea; ^2^ Department of Materials Science and Engineering Korea Advanced Institute of Science and Technology Daejeon 34141 Republic of Korea

**Keywords:** coffee‐ring, confinement effect, polygonal quantum dot light‐emitting diodes, solutal marangoni effect, vertex, vortex pair

## Abstract

Currently, quantum dot light‐emitting diodes (QD‐LEDs) are receiving extensive attention. To maximize their luminous performance, the uniformity of the QD‐LEDs is crucial. Although the spontaneously self‐induced solutal Marangoni flow of an evaporating binary mixture droplet has been widely investigated and used to suppress coffee‐ring patterns in ink‐jet printing technology, unfortunately, ring shapes are still present at the edges, and the Marangoni flow generated by the selective evaporation of volatile liquid components cannot be controlled due to its nonlinear instabilities. In this work, polygonal coffee‐ring‐less QD microarrays are created using two spontaneous and sequential solutal Marangoni flows. During the initial evaporation, internal circulating flows are controlled by polygonal‐shaped droplets. After that, sequential interfacial flows are generated by the captured volatile vapors. A theoretical model and scaling analysis are provided to explain the working mechanisms. It is expected that the newly designed printing system can be applied to the mass production of QD‐LEDs.

## Graphical abstract

Evaporatively self‐induced sequential Marangoni flows yielded a coffee‐ring‐less polygonal shapes of quantum dot microarrays.

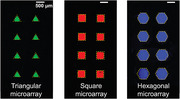

## 1 Introduction

Colloidal quantum dots (QDs) are excellent semiconducting nanomaterials with great potential due to their emission wavelengths being tunable with the QD size and their narrow light emission peak, which can contribute to high color saturation and better luminous efficiency. Through these advantages, they can be widely utilized in quantum dot light‐emitting diodes (QD‐LEDs),^[^ ^1^, ^2^, ^3^ ^]^ solar systems^[^ ^4^, ^5^ ^]^ with improved DC conversion efficiency, and biological tools for medical diagnosis.^[^ ^6^ ^]^ In particular, in the display industry field, quantum dots are of interest as alternatives to organic light‐emitting diodes (OLEDs),^[^ ^7^ ^]^ as quantum dots are heat‐resistant inorganic materials with long lifespans. To date, relevant studies have suggested methods for obtaining uniform QD patterns to maximize their luminous intensity by using several surface‐coating methods: transfer printing,^[^ ^8^, ^9^ ^]^ photoresist patterning,^[^ ^10^ ^]^ and ink‐jet printing.^[^ ^11^, ^12^ ^]^ Among them, the transfer printing and photoresist patterning require pressing and heating processes, respectively, which physically damage the quantum dots themselves. Therefore, the ink‐jet printing technique is considered the most ideal surface‐coating method for producing uniform QD films due to its low waste of quantum dots and streamlined processes (i.e., simple evaporation).^[^ ^13^ ^]^ In ink‐jet printing systems, QD deposition patterns with various and complicated shapes^[^ ^14^ ^]^ are receiving much attention due to their potential to improve the display resolution and aperture ratio (i.e., the area ratio of the part that can emit light in a unit pixel) by changing the red‐green‐blue pixel arrangements.^[^ ^15^ ^]^ However, most preceding studies have mainly focused on a simple geometry, for example, circular^[^ ^12^ ^]^ and square^[^ ^16^ ^]^ configurations and evaporation mechanisms of the spherical droplets so far.^[^ ^17^, ^18^ ^]^ Hence, in this work, we intensively focus on examining uniform QD films with various and more complicated morphologies, such as triangular and hexagonal configurations. In a previous study, it was reported that the coffee‐ring effect^[^ ^19^ ^]^ can be accelerated if a droplet has a relatively larger local curvature along a contact line, in which a larger amount of the coating materials is ultimately piled up at the high curvature region. Thus, controlling and suppressing the coffee‐ring phenomenon in polygonal droplets is essential for achieving uniform polygonal QD patterns in ink‐jet printing systems. Here, we introduce coffee‐ring‐less polygonal QD films using simple evaporation in a confined space, where self‐induced sequential Marangoni effects occur; 1) side‐to‐vertex circulating solutal Marangoni flow is generated by relative evaporation between the vertex and the side of the polygonal droplets and 2) the confined geometry can induce long‐lasting solutal Marangoni flows directed radially inward driven by evaporated volatile components.^[^ ^20^, ^21^ ^]^ Using particle image velocimetry (PIV), we demonstrate that internal flow opposite that of the coffee‐ring effect is generated when the polygonal multicomponents droplet evaporates in the confined space, and finally, we obtain coffee‐ring‐less QD red/green/bule (R/G/B) microarray patterns using the confinement effect without any complicated drying processes or chemical additives. Furthermore, we validate that the flow structures inside the polygonal binary mixture drop can be controlled by the number of vertices (triangle: three vertices, square: four vertices, and hexagon: six vertices) where phase segregation^[^ ^22^, ^23^ ^]^ occurs at the vertex and side because each has a different contact angle. This implies that the internal flows inside the binary mixture drop can be controlled by the geometry of the droplet although it is known that a conventional hemispherical drop has unpredictable and complicated mixing flows during evaporation with binary components.^[^ ^24^ ^]^ Additionally, we suggest a scaling argument and theoretical model to explain the flow control inside binary polygonal droplets by governing the number of vertices as a valuable parameter.

## 2 Results and Discussion

### 2.1 Polygonal Coffee‐Ring‐Less Dried Patterns for QD Microarrays

**Figure 1:**
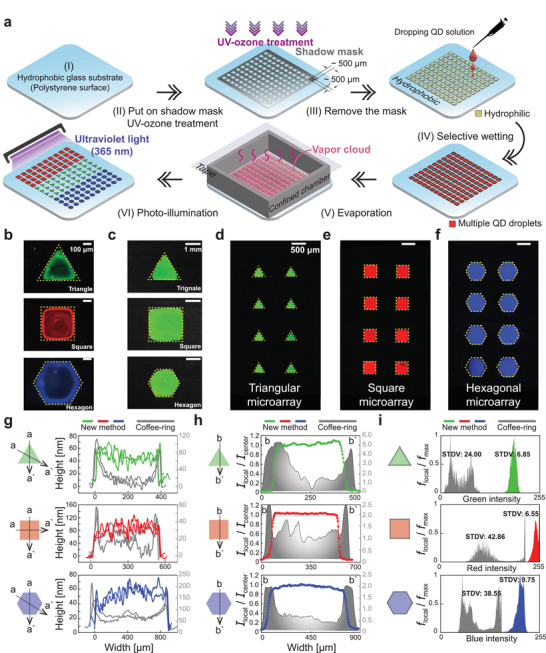
Multiarray uniform quantum dots patterns. a) Fabrication processes for micro QD array. b) QD micro‐size coffee‐ring polygonal patterns after QD binary mixture drops evaporate. The scale bars are 100 µm. c) A millimeter size green QD polygonal patterns. The scale bars are 1 mm. d–f) QD micro‐size‐arrays with triangular, square, and hexagonal patterns. The scale bars are 500 µm. The yellow‐dashed lines represent contact lines. g) Comparison of height profiles for the triangular, square, and hexagonal patterns along a–a′ lines. h) Comparison of photoluminescence profiles normalized with the light intensity at the center *I*_center_ along b–b′ line. i) Comparison of R/G/B image histograms with a relative frequency value *f*_local_/*f*_max_.

**Figure** **1**a illustrates a fabrication process for producing coffee‐ring‐less polygonal QD microarrays: I) preparation of superhydrophobic substrate with polystyrene (PS), II) UV–ozone cleaning and treatment using a shadow mask on the substrate, III) removal of the mask, IV) discharging of QD bulk solution and selective wetting of the droplet, V) generation of evaporation and the solutal Marangoni effect, and VI) illumination of the final dried QD patterns using UV light. From the above surface‐coating processes, as shown in Figure 1c–f, we ultimately obtain uniform polygonal QD arrays on the order of 100 µm to 1 mm simply using evaporation of multiple QD binary mixture drops composed of DI water and ethanol in a confined chamber, while QD binary mixture droplets in an open space leave coffee‐rings (see Figure 1b). Here, the polygonal QD patterns with triangular, square, and hexagonal shapes exhibit great photoluminescence uniformity. In Figure 1g, a height measurement is conducted to compare its deposit uniformity with that of the conventional coffee‐ring QD patterns (Figure 1b), which shows that our newly proposed QD printing method can perfectly suppress the coffee‐ring effect at the edges of the polygonal droplet. Additionally, we confirm that much better uniformity of photoluminescence profiles is observed in our QD pattering results in Figure 1h. In the image R/G/B histogram analysis in Figure 1i, the spatially confined ink‐jet system leads to much narrower and stronger high intensity peaks with lower standard deviations (STDV) (triangle: 6.85, square: 6.55, and hexagon: 9.75) compared with the coffee‐ring results (triangle: 24.00, square: 42.86, and hexagon: 38.55), where the QD coating pattern has a better luminous uniformity as the peaks become narrower and sharper.

### 2.2 Coffee‐Ring Effect Acceleration by Vertices in Polygonal Drops

**Figure 2:**
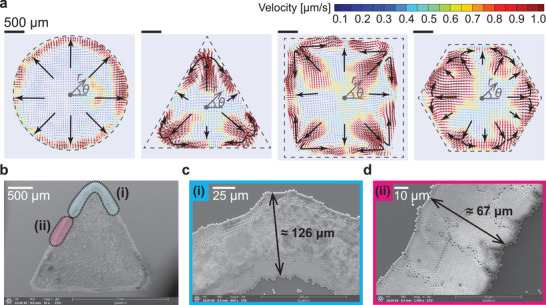
Coffee‐ring flows and particle packing structures in polygonal drops. a) Particle image velocimetry (PIV) results for a pure water droplet on an open substrate just before evaporation is complete (Here, the flow fields are measured at a different time, hemispherical: *t* ≈ 780 s, triangular: *t* ≈ 420 s, square: *t* ≈ 900 s, and hexagonal: *t* ≈ 600 s.). Black arrows indicate representative flow patterns inside the liquid droplet and dashed‐gray lines show initial contact lines. In the middle of the wetted area, we set a (*r*, *θ*) polar coordinate. The scale bars are 500 µm. b–d) Scanning electron microscope (SEM) top view images of a dried pattern of triangular droplets consisting of the pure water. b) A SEM top view for a triangular droplet. c–d) Particle packing structures around i) vertex and ii) side of (b), respectively.

In a previous study,^[^ ^25^ ^]^ using a numerical tool, it was proven that a polygonal droplet shows an asymmetric evaporative flux *J* along the radial and azimuthal directions owing to its nonconstant curvature along a contact line, as shown in Figure S1c, Supporting Information. As a result, coffee‐ring flow, that is, evaporation‐driven capillary flow,^[^ ^26^ ^]^ toward the vertices, becomes much stronger than that of a conventional hemispherical drop. If a polygonal droplet of single liquid component evaporates, the coffee‐ring flows form toward the vertices because of the large amount of volume loss caused by the locally higher evaporative fluxes at the vertices and are replenished from bulk fluid based on volume conservation. To further validate this hypothesis, we performed experiments with polystyrene particles at a 5.0% v/v concentration. In **Figure** **2**a, we observe and compare the coffee‐ring flows inside polygonal drops, such as triangular (three vertices), square (four vertices), and hexagonal (six vertices) drops, depending on the number of vertices with the conventional hemispherical drops by using PIV measurements. From the above results, we confirm that, if vertices are present in the droplet, the coffee‐ring effect becomes more serious around all vertices. Additionally, side‐to‐vertex corner flows are observed due to residual forces driven by the dominant capillary flows toward the vertices (see Supplementary Movies 1, 2, 3, and 4). Consequently, as shown in Figure 2b–d, more polystyrene particles inside the polygonal drop are piled up around all the vertices where the area in the vicinity of the vertex has approximately two times thicker packing structures than the side of the wetted area.

### 2.3 Vertex Effect in an Evaporating Binary Mixture Drop

**Figure 3:**
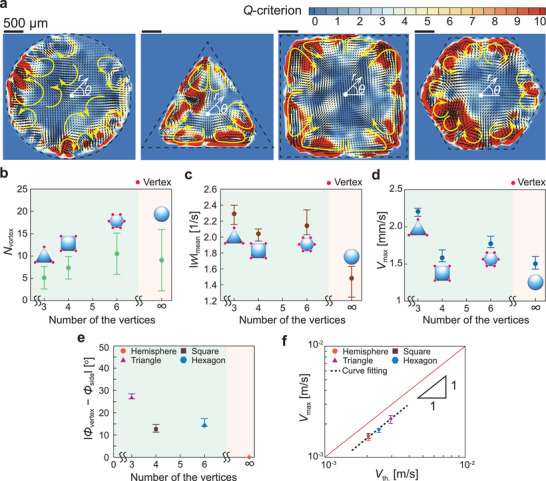
Experimental and theoretical results for flow fields in binary mixture drops. a) Flow field results for an ethanol–water mixture (50 : 50 vol.%) droplet on an open substrate from PIV measurements. We observed flow patterns for all the cases when the internal flows become a quasi‐state after the evaporation starts (*t* < 4 s). Yellow arrows represent the typical flow patterns. A polar coordinate (*r*, *θ*) is set in the middle of the wetted area. The scale bars indicate 500 µm. b) Comparison of the number of representative vortices *N*_vortex_ depending on a different drop shape. Here, the greenish and radish area indicate polygonal drops and hemispherical drops, respectively. c) Comparison of mean vorticity ${\left|W\right|}_{\mathrm{mean}}={\left|\nabla \times \vec{V}\right|}_{\mathrm{mean}}$ where $\vec{V}=\left(u_{1},u_{2}\right)$ in different drop shapes. d) Comparison of maximum velocity *V*_max_ for different drop shapes. The pink dots indicate the vertex of the polygon. e) Contact angle difference Δ*ϕ*_c_ = |*ϕ*_vertex_ − *ϕ*_side_| between the vertex and side. The error bars were obtained from multiple measurements. f) Comparing theory and measurement for *V*_max_.

To suppress the coffee‐ring effect for quantum dot displays, a dual‐solvent system^[^ ^27^, ^28^ ^]^ has been widely used as one of the uniform QD coating methods thus far, where Marangoni flows are generated by selective evaporation^[^ ^22^, ^23^ ^]^ along a liquid–gas interface. Here, the mixing flows driven by the Marangoni effect can help to obtain uniform QD films, but it is almost impossible to control them due to the nonlinearity of complex flows.^[^ ^24^, ^25^ ^]^ In this work, we notice that the complicated flows inside the binary mixture drops can be controlled by the vertex structure of a polygonal drop from PIV measurements, as shown in **Figure** **3**. In Figure 3a and Movies S5–S8, Supporting Information, side‐to‐vertex circulating vortex pairs are generated at the vertices during evaporation of the polygonal drops, whereas a hemispherical drop shows complicated mixing flows^[^ ^24^ ^]^ (here, ethanol: *γ* ≈ 22.39 mN⋅m^−1^ and water: *γ* ≈ 72.8 mN⋅m^−1^). As shown in Figure S1c, Supporting Information, the vertex shows a higher evaporative flux and then has a lower ethanol concentration. Here, we can neglect the evaporation of the water because ethanol is much more volatile than water (ethanol: *P*_v_ ≈ 5.95 kPa and water: *P*_v_ ≈ 2.34 kPa). Finally, a higher surface tension *γ*_high_ is developed at the vertex. In contrast, the side has a lower surface tension *γ*_low_ because of the same mechanism. As a result, the circulating internal flows are observed around all the vertices in the polygonal drops from the side to the vertex. Thus, we realize that it is possible to control the internal flow patterns in the binary component liquid droplet using selective evaporation between the vertices and sides. The controlled circulating internal flows by the vertices are investigated further in this paragraph. The circulating flows contribute to mixing the suspended coating materials in the early stage. To detect the vortices, we use the *Q*‐criteria method,^[^ ^29^ ^]^ which is one of the general methods for investigating vortex structures, by analyzing the rates of rotation $\Omega_{\mathrm{ij}}=\;\frac{1}{2}\left(\frac{\partial u_{i}}{\partial x_{j}}-\frac{\partial u_{j}}{\partial x_{i}}\right)$ and strain $S_{\mathrm{ij}}\;=\;\frac{1}{2}\left(\frac{\partial u_{i}}{\partial x_{j}}+\frac{\partial u_{j}}{\partial x_{i}}\right)$ from the velocity gradient tensor $L_{\mathrm{ij}}=\frac{\partial u_{i}}{\partial x_{j}}=\Omega {\;}_{\mathrm{ij}}+S_{\mathrm{ij}}$, where i, j = 1, 2 in 2D Cartesian coordinates, expressed as follows

(1) $$Q=\frac{1}{2}\left(∥\Omega_{\mathrm{ij}}{∥}^{2}-∥S_{\mathrm{ij}}{∥}^{2}\right)$$

Here, *Q* > 0 shows the existence of a vortex and the magnitude of *Q* indicates the strength of the vortex in a flow field. Figure 3a shows that the hemispherical drop has weaker vortices irregularly distributed in the flow field, whereas in the case of polygonal drops, strong vortices are observed around all vertices. Additionally, we count the number of representative vortices *N*_vortex_ for all the cases using a *λ*_2_ criterion^[^ ^30^ ^]^ (see Experimental Section). Consequently, we find that a pair of *N*_vortex_ values almost equal to the number of vertices in the polygonal droplets in Figure 3b. For the hemispherical drops, we observe that there are no typical vortex structures due to nonlinear selective evaporation (see also Movie S5, Supporting Information).^[^ ^31^ ^]^

To uniformly mix the suspended particles in an evaporating droplet, it is important to have strong circulating flows. We compare the maximum velocity *V*_max_ and mean vorticity |*W*|_mean_ for a hemispherical and polygonal droplet with an ethanol–water mixed solution (see Figure 3c,d). We observe that the triangular droplet has the largest maximum velocity and mean vorticity, followed by the hexagonal, square, and circular droplets. There are two main reasons for this result: One is that the largest vertex‐and‐side contact angle difference is found in the triangular droplet in Figure 3e. As the contact angle difference increases, the gradient of the evaporative flux along the azimuthal direction (e.g., between a vertex and side) Δ*J* = ∣*J*_vertex_ − *J*_side_∣ becomes larger. Then, finally the surface tension gradient ∣Δ*γ*∣ = ∣*γ*_vertex_ − *γ*_side_∣ simultaneously becomes larger.^[^ ^21^, ^22^, ^23^, ^24^ ^]^ The other reason is that less momentum exchange by the vortices occurs in the triangular drop due to its relatively larger and fewer vortices. As the number of vertices increases, the number of vortices increases, and the interaction between vortices becomes vigorous. For the circular droplet case, the vortices occur randomly and are crowded. The momentum exchange can be estimated by viscous effects with a viscous coefficient *μ* and we can compare the momentum diffusion time scale by vortices (≈*ρ* *L* ^2^ *μ* ^−1^) for all cases, where *L* is the vortex size. Consequently, the triangular drop has the largest momentum diffusion time scale, that is, minimum momentum loss. Therefore, the triangular droplet has the strongest velocity and vorticity fields.

### 2.4 Theoretical Model for Controlled Internal Flows of Polygonal Binary Mixture Drops in an Open System

We showed that the number of vertices can control the internal circulating flows. To understand and explain the working mechanism, we try to develop a theoretical model for the maximum velocity *V*_max_ near the corner (or vertex) of the polygonal droplet. To perform a scaling argument, we use some assumptions corresponding to our experimental measurements as follows: i) the gravity effect is negligible due to *Bo* =  Δ*ρ* *gl*/*γ* ≪ 1, ii) the viscosity effect is slightly more dominant than inertia, but it is also comparable due to *Re* = *ρ* *Vl*/*μ* ⩽ 1, iii) the Marangoni effect is predominant compared to diffusion due to *Ma* = ∣Δ*γ*∣*l*/*μ* *D* ≫ 1, and iv) the relative surface tension gradient between the vertex and side is considered a correction factor $\alpha (=1\;+\;\Delta \phi_{c}^{*})$. The correction factor is dependent on the normalized contact angle difference $\Delta \phi_{c}^{*}=|\phi_{\mathrm{vertex}}-\phi_{\mathrm{side}}|/\Delta \phi_{\max}=|\Delta \phi_{c}|/\left(\pi /2\right)$, where Δ*ϕ*_max_ is the maximum contact angle difference; *l* is a representative length (here, a droplet radius) of $∼\sqrt{V_{o}/h_{o}}$, *V*_o_ is the initial droplet volume; *ρ* is the density (≈ 900 kg⋅m^−3^); *g* is the gravity acceleration (≈ 9.8 m⋅s^−2^); Δ*γ* is the surface tension gradient (≈ 10^−2^ N⋅m^−1^); *μ* is the dynamic viscosity (≈ 0.003 Pa⋅s); and *D* is the diffusion coefficient of an ethanol–water mixture (≈ 0.4 × 10^−9^ m^2^⋅s^−1^).^[^ ^32^ ^]^ Here, we assume a steady flow structure. With the above assumptions, we estimate solutal Marangoni flows between the vertex and side where the control volume is the droplet. Then, scaling modeling starts from steady energy conservation Equation (2)

(2) $${\left(\dot{Q}-\dot{W}\right)}_{\mathrm{in},\mathrm{net}}=\int_{\mathrm{cs}}\left(u+pv+\frac{V^{2}}{2}+gz\right)\rho \vec{V}\cdot d\vec{A}$$

where $\dot{Q}$ is an in/out heat flux (J⋅s^−1^), $\dot{W}$ is an in/out work flux (J⋅s^−1^), *U* is the specific internal energy (J⋅kg^−1^), *p* is the pressure (kg⋅m^−1^⋅s^−2^), *v* is the specific volume (m^3^⋅kg^−1^), *V* is the fluid velocity (m⋅s^−1^), *gz* is the gravitational work per unit mass (J⋅kg^−1^), and d*A* is the unit surface area (m^2^). To simplify this problem, we suppose that thermal effects are almost negligible during evaporation because the solutal Marangoni effects are predominant^[^ ^23^, ^24^, ^33^ ^]^ and neglect the enthalpy variation Δ*h* [=Δ(*U* + *pv*)] where *U* is an internal energy, which is a function of only temperature in a liquid phase. Additionally, we presume that evaporation goes through an adiabatic process from the surrounding environment. Finally, the dynamic energy ${\dot{E}}_{d}$ is generated by balancing the surface tension energy ${\dot{W}}_{\mathrm{surf}.}$ induced by the Marangoni stress and the viscous dissipation energy ${\dot{W}}_{\mathrm{visc}.}$. Then, we can obtain

(3) $$\;-{\dot{W}}_{\mathrm{visc}.}+{\dot{W}}_{\mathrm{surf}.}\approx {\dot{E}}_{d}$$

where ${\dot{E}}_{d}$ is $\frac{1}{2}\rho V^{3}A$. For the three remaining terms in Equation (3), we estimate the energy scale as

(4) $${\dot{W}}_{\mathrm{visc}.}=\int \mu {\left(\nabla V\right)}^{2}dV_{o}\approx \mu {\left(\frac{V_{\mathrm{th}.}}{h_{o}}\right)}^{2}V_{o}$$

(5) $${\dot{W}}_{\mathrm{surf}.}\approx \frac{\alpha |\Delta \gamma |h_{o}^{2}}{t_{\nu }}\approx \frac{\mu \alpha |\Delta \gamma |h_{o}^{2}}{\rho h_{o}^{2}}=\frac{\mu \alpha |\Delta \gamma |}{\rho }$$

(6) $${\dot{E}}_{d}\approx \frac{1}{2}\rho V^{3}A∼\rho {\left(V_{\mathrm{th}.}\right)}^{3}\pi \left(R^{2}+h_{o}^{2}\right)$$

where *V*_th._ is the velocity from theory. Here, we use the viscous time scale (*t*_ν_ $\;∼\rho h_{o}^{2}\mu^{-1}$) in ${\dot{W}}_{\mathrm{surf}.}$ in that the viscous effect is slightly more dominant in this problem based on *Re* ⩽1. The whole surface area *A* is replaced by $\pi (R^{2}+h_{o}^{2})$ assuming that the droplet shape in all cases is similar to a spherical cap. If substituting the above energy scaling Equations (4)–(6) into Equation (3), we have

(7) $$\;-\mu {\left(\frac{V_{\mathrm{th}.}}{h_{o}}\right)}^{2}V_{o}+\frac{\mu \alpha |\Delta \gamma |}{\rho }\approx \rho {\left(V_{\mathrm{th}.}\right)}^{3}\pi \left(R^{2}+h_{o}^{2}\right)$$

where *α* varies depending on $\Delta \phi_{c}^{*}$, which is determined by the *N*_vertex_ of the evaporating drops. In the case of ∣Δ*γ*∣, we can assume that ∣Δ*γ*∣ might be of the order of 0.1 mN⋅m^−1^. In a previous study, for Marangoni flows, the upper bound for ∣Δ*γ*∣ was estimated to be 1 mN⋅m^−1^ if there was negligible deformation of the liquid–gas interface^[^ ^33^ ^]^ and ∣Δ*γ*∣  ≈  1 mN⋅m^−1^ would generate a typical velocity of 1 mm⋅s^−1^.^[^ ^34^ ^]^ Our experimental velocity results show an average velocity on the order of 0.1 mm⋅s^−1^. Finally, we calculate *V*_th._ from Equation (7) and compare it with *V*_max_ (see Figure 3f). We confirm that the theoretical model proposed in this study explains the controllable internal flow in the binary mixture drop with the polygonal shape well.

### 2.5 Vapor‐Driven Interfacial Solutal Marangoni Flows via the Confinement Effect

**Figure 4:**
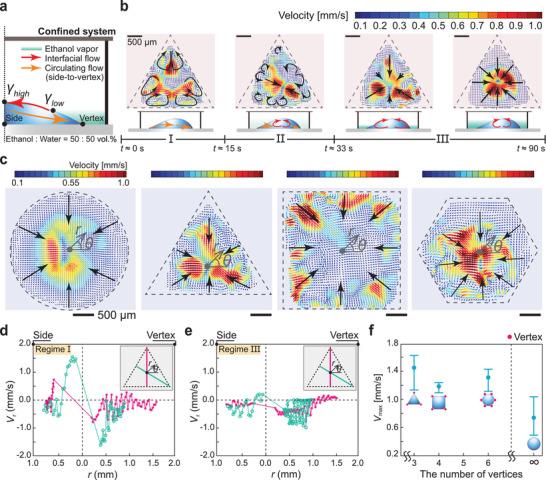
Flow fields in a confined system. a) Schematics of two spontaneous and sequential solutal Marangoni effects in a confined condition. An orange and red arrow indicate a typical flow during early and mid‐late evaporation, respectively. b) Time evolution of the flow fields inside a triangular ethanol–water mixture (50 : 50 vol.%) drop on a substrate in a confined chamber, which is classified as follows: regime I, controlled side‐to‐vertex circulating flows at all the vertices (same as an open system), regime II, transition of the Marangoni flow structures, and regime III, radially inward interfacial flows. A schematic of the side view is provided below each regime. c) Flow field results for all different shapes of the ethanol–water mixture droplets in the confined chamber (Here, 30 s ⩽ *t* ⩽ 110 s). Black arrows indicate typical flow patterns and dashed‐gray line shows an initially wetted area. Here, we set polar coordinates (*r*, *θ*) as (c). All the scale bars are 500 µm. A radial velocity *V*_r_ is investigated along the azimuthal direction for d) regime I and e) regime III at *θ* = 90° and *θ* = 150°. Here, (*r*, *θ*) coordinates are set at the centroid position of each geometry (black dots). The color of velocity vectors in the flow field indicate the magnitude of the velocity. Here, magenta and light‐green solid lines represent *θ* = 90° and *θ* = 150°, respectively. f) Comparison of maximum velocity *V*_max_ for all different shapes of the droplets in the confined condition.

In this work, we deposit a polygonal droplet in a confined system as shown in Figures 1a and **4**a. The droplet evaporates over time, and two different solutal Marangoni flows are observed that occur spontaneously and sequentially. Figure 4b describes the time‐dependent flow transition of a triangular droplet during evaporation in the confined geometry.

We observe that, in the early time period, the strong side‐to‐vertex circulating flow in a binary mixture drop is induced and controlled by the number of vertices, and it helps to mix the coating materials in the evaporating drop. Moreover, to totally suppress the coffee‐ring effect, we need to generate an additional spontaneous and sequential Marangoni effect even after the circulating internal flow driven by selective evaporation between a vertex and side weakens over time. After a few seconds, the regular vortices near the vertex become weaker, and other interfacial flows occur (regime II). In regime III, vapor‐driven solutal Marangoni effects from captured volatile vapors in a confined chamber become predominant (see Figure 4a,b). If a multicomponent liquid droplet evaporates in the confined space where all the liquid solvents have a different surface tension and molecular weight from those of the more volatile component which is greater than that of ambient air (*M*_air_ ≈ 28.84 g⋅mol^−1^), initially, a flow pattern similar to that of an open system occurs (see the orange arrow of Figure 4a and regime I of Figure 4b), and then volatile vapors (here, ethanol) stagnated near the interface of the evaporating drop gradually induce solutal Marangoni flows in the radially inward direction opposite the coffee‐ring flow along the liquid–gas interface (see the red arrow of Figure 4a and regimes II and III of Figure 4b).^[^ ^21^ ^]^ Additionally, we verify that, regardless of droplet geometries, for example, triangular, square, and hexagonal configurations, long‐lasting interfacial flows toward the center of the drop occur and they become dominant during evaporation in the confined system (see Figure 4c and Movies S9–S12, Supporting Information). Furthermore, to compare two sequential flows (regimes I and III), we analyze a radial velocity profile along the radial and azimuthal directions in Figure 4d,e. In the case of regime I, at *θ* = 90° and *θ* = 150° in polar coordinates (*r*, *θ*), the positive radial velocity +*V*_r_ (outward flow) and negative radial velocity −*V*_r_ (inward flow) from the origin (0,0) are observed near the side and vertex, respectively (see Figure 4d). However, in regime III, negative flows −*V*_r_ converging at the origin (0,0) are only observed (see Figure 4e).

In Figure 4f, we also show that the confinement effect is much more effective when the polygonal binary mixture drops evaporate in the confined space. If a vertex structure is present, stronger velocity fields are observed during confined evaporation. The reason is that driving forces from the side‐to‐vertex circulating internal flow make them much stronger than those in a conventional hemispherical drop. As the evidence of above facts, a triangular droplet has the maximum velocity along the droplet surface, followed by hexagonal, square, and hemispherical droplets, in that sense, the strongest inward circulating flow around all the vertices is shown in the triangular case (see Figure 3c,d). Finally, we demonstrate that follow‐up solutal Marangoni flows by the confinement effect can contribute to achieving polygonal‐shaped QD arrays with totally suppressed coffee‐ring stains.

## 3 Conclusion

In this work, we developed a novel coating method to achieve polygonal coffee‐ring‐less QD microarrays that could be applicable for mass production in ordinary environmental conditions. To avoid complicated processes and technologies,^[^ ^8^, ^9^, ^10^ ^]^ we designed the patterning system of a polygonal droplet of QD solution. In the method newly proposed in this study, two sequential Marangoni flows are self‐induced by selective evaporation of a binary mixture due to the geometry of the droplet and stagnant volatile vapors in a confined chamber during evaporation. In the early stage, vertices in the polygonal drops can control internal circulating flows and strengthen velocity and vorticity fields; these are the solutal Marangoni flows driven by the relative evaporative flux between the vertex and side. This circulating flow provides a homogeneous QD distribution during the early evaporation stage. Then, if we capture the evaporated vapors of the binary mixture near the evaporating droplet, we can generate interfacial Marangoni stresses that totally suppress the coffee‐ring pattern, which is a vapor‐driven solutal Marangoni effect. Two different Marangoni effects occur spontaneously and sequentially, which completely suppress the accumulation of nano‐ and micro‐particles at the contact line during evaporation. We also showed that this method can fabricate coffee‐ring‐less dried patterns with millimeter and micrometer polygonal shapes. The current method guarantees safe, easy, low‐cost, and large format quantum dot light‐emitting diodes (QD‐LEDs) fabrication. We expect that, if a commercial large‐format ink‐jet printing system adopts this method on predesigned surface patterns with high surface energy contrast, it would overcome the complexity and limitations of the current mass production technology with the better uniformity of QD multiarrays.

## 4 Experimental Section

### Materials

Water‐soluble quantum dots [red: CZM‐620W (size: 12.0 ± 1.5 nm), green: CZM‐530W (size: 10.0 ± 1.5 nm), and blue: CZM‐450W (size: 9.0 ± 1.5 nm)] with a ligand thickness 6 to 9 nm were purchased from ZEUS (South Korea). The QDs were stably dispersed in the pure water solutions with a particle concentration of 5 mg⋅mL^−1^ and *ζ*‐potential −35 to −40 mV in Figure S2, Supporting Information.^[^ ^35^ ^]^ Ethanol was used with ⩾99.5% purity from Sigma‐Aldrich (USA) and water distilled by a ultraviolet water purification device (Direct‐Q3 UV) from Sungwoo Genetech (South Korea). QD coating solutions were made with a mixture, ethanol : water : QDs = 48.6 : 51.1 : 0.3 wt% where the QD particles in the solution were stable with *ζ*‐potential −20 to −25 mV (see Figure S2, Supporting Information). 3D printed confined chamber was manufactured by using 3D printer (3DWOX 1) produced from Sindoh (South Korea) with polylactic acid (PLA) materials. To perform a hydrophilic treatment, a plasma treatment gun (BD‐10ASV) from Electro‐Technnic Products, INC., (USA) and UV–ozone cleaner (AC‐3) from AhTech LTS (South Korea) were used. The PDMS shadow mask was made with a weight ratio; silicon elastomer base:silicon elastomer curing agent = 10 : 1 wt% purchased from DOW (USA). For PIV experiment, fluorescent micro‐particles (PS‐FluoRed‐Fi320) were purchased from GmbH (Germany) and high‐speed camera (Mini‐AX200) manufactured by Photron (Japan) were used to observe flow structures inside the droplet.

### Fabrication of Quantum Dots Multi Microarrays

As shown on Figure 1a, the process follows: I) Superhydrophobic covered by polystyrene (PS) brush (35,000 g·mol^−1^) and a stainless steel shadow mask with 150 × 150 × 0.2 mm^3^ size were prepared. II) UV‐ozone treatment was done on the glass substrate covered by the shadow mask with about 500 µm polygonal multiple holes to generate surface free energy difference between an uncovered and covered part. Consequently, the surface uncovered by the mask became more hydrophilic. III) After getting rid of the shadow mask, QD bulk‐solution were discharged on the whole substrate. IV) The discharged QDs solution was spread and selectively deposits only on the hydrophilic area. As a result, V) Well‐aligned multiarray QD droplets were produced and evaporated in a confined chamber fabricated by 3D printer. As time goes, the evaporated volatile vapors were captured by the confined boundary and continuously generated solutal Marangoni effect causing radially‐inward interfacial flows. VI) Ultimately, images of polygonal coffee‐ring‐less QD microarrays illuminated by a UV light with a wavelength *λ* = 365 nm were obtained.

### QD Film Thickness Measurement

The thickness profiles of a polygonal QD microarray film were measured by using a laser scanning confocal microscope (VK‐X1050, Keyence, Japan) which can provide a maximum 5 nm height‐resolution with a high magnification in Figure 1g. Through image stitching technique, a scanning area (600 × 600 mm^2^) was set and its height profiles were measured with a 10× magnification.

### Photo‐Luminescence Evaluation

Using ImageJ tool,^[^ ^36^ ^]^ the straight‐line luminescence profiles were measured. To compare a luminous uniformity with a conventional coffee‐ring pattern with 500 µm size, the local light intensity *I*_local_ was normalized with the light intensity at the center *I*_center_ from the resulting data, as shown in Figure 1h. Moreover, image histogram results for all the 500 µm polygonal depositions (green: triangle, red: square, and blue: hexagon) between an open and confined system (Figure 1i) were analyzed and compared. Here, all the local frequency data *f*_local_ were divided by the maximum frequency *f*_max_.

### Polygonal Droplet Generation

To create polygonal liquid droplets, a plasma treatment was performed on a PDMS shadow mask with polygonal‐shaped holes produced by a steel shape puncher, for example, triangle, square, and hexagon. Here, an uncovered surface area became more hydrophilic. After the plasma treatment, the droplet was discharged using a micro‐pipette (triangle: 1.0 ± 0.02 µL, square: 2.40 ± 0.05 µL, hexagon: 1.50 ± 0.03 µL, and circular: 2.0 ± 0.04 µL, respectively) and ultimately deposited on the hydrophilic treated surface due to the difference in surface free energy, as shown in Figure S1a, Supporting Information. Here, the discharged initial volume was determined by matching a constant ratio *V*_o_/*A*, where *V*_o_ was an initial droplet volume and *A* was a wetted area that was controlled by inscribed in a 3 × 3 mm^2^ square in Figure S1b, Supporting Information. Actually, the initial heights *h*_o_ for all the droplets were measured as ≈ 0.37 mm.

### Experimental Set‐Up for PIV

In the PIV experiments (Figure S1d, Supporting Information), two solutions were prepared; distilled (DI) water and binary mixture containing ethanol and DI water with a volume ratio 50 : 50 vol.% that was widely utilized in a drying pattern study.^[^ ^26^ ^]^ To visualize internal flows inside the polygonal droplets using PIV, fluorescent particles with a diameter of 1.90 ± 0.09 µm and 2.5% w/v concentration were added into both pure water and binary mixture solutions. The particles were uniformly dispersed with *ζ*‐potential −20 to −30 mV.^[^ ^35^ ^]^ Here, the particle concentration was 1% v/v in all the solutions. After that, they were deposited on a cover glass and evaporated at room temperature of 20 °C. Image sequences of illuminated fluorescent particles were recorded using high‐speed camera with 50 frames s^−1^ (for a pure water) or 250 frames s^−1^ (for a binary mixture). Here, the droplet height *h*_o_ was much smaller than the droplet radius *R* (i.e., *h*_o_/*R*  < 1). Therefore, the focal plane was set to the vicinity of the bottom part of the droplet where the depth of field was about ≈ 150–200 µm from the surface of the cover glass. Flow field calculation was conducted by PIVlab tool in MATLAB.^[^ ^37^ ^]^ To obtain vector fields, iterative 2D cross‐correlation of the particle images was applied with multiple interrogation windows of 64 × 64 pixels (first) and 32 × 32 pixels (second) with 50% overlaps where the signal‐to‐noise ratio (SNR >3) was satisfied for the reliable PIV.^[^ ^38^ ^]^ Here, all suspended particles would well follow the Marangoni convective flows in that Stokes number ($St=\frac{\rho_{p}d_{p}^{2}u_{o}}{18\mu_{l}R}$) is much smaller than unity where *ρ*_p_ is the particle density, *d*_p_ is the particle diameter, *μ*_l_ is the dynamic viscosity of the solvent, *u*_o_ is the flow speed, and *R* is the droplet radius. During PIV measurements, a liquid droplet was covered with an acrylic box to prevent external forces from affecting the internal flows inside the droplet. In the case of confined systems, the droplet was covered by a 3D printed chamber (10 × 10 × 5 mm^3^) with diameter 6 mm hole during the evaporation, as shown in Figure S1d, Supporting Information.

### Vortex Detection

Rotation rate tensor $\Omega_{ij}=\;\frac{1}{2}\left(\frac{\partial u_{i}}{\partial x_{j}}-\frac{\partial u_{j}}{\partial x_{i}}\right)$ and strain rate tensor $S_{ij}=\;\frac{1}{2}\left(\frac{\partial u_{i}}{\partial x_{j}}+\frac{\partial u_{j}}{\partial x_{i}}\right)$ were calculated, where i, j = 1, 2, using PIV results conducted by PIVlab tool in MATLAB. After that, the precaculated data were substituted into *Q*‐criterion Equation (2) which can be expressed as $\frac{1}{2}\left(\Omega_{ij}\Omega_{ij}-S_{ij}S_{ij}\right)$, where i, j = 1, 2. As a result, information of the vortex trace in flow fields was obtained by iterating the above calculation processes. Also, how many the typical vortices were observed in a hemispherical and polygonal drop were counted and compared by calculating $\lambda_{2}=\mathrm{tr}{\left(X\right)}^{2}-4\times \det\left(X\right)$, where $X_{\mathrm{ij}}=\frac{\partial u_{i}}{\partial x_{j}}$ (i, j = 1, 2).^[^ ^30^ ^]^ Among all the calculation results, minimum *λ*_2_ peaks were extracted with 25% threshold set to ignore negligible small vortices.

### Statistical Analysis

A velocity field inside an evaporating liquid droplet was measured using an open‐source MATLAB library PIVlab.^[^ ^37^ ^]^ In the postprocessing, spurious data (i.e., outliers) in a superimposed velocity scatter plot were identified and neglected where the unexpected data had a large fluctuation with respected to a global mean. Also, results of the vorticity, *Q*‐criterion and *λ*_2_ had a standard deviation of about 0.014 which arose from calculations of the flows in the out‐of‐plane direction.^[^ ^38^ ^]^

## Conflict of Interest

The authors declare no conflict of interest.

## Supporting information

Supporting Information

Supplemental Movie 1

Supplemental Movie 2

Supplemental Movie 3

Supplemental Movie 4

Supplemental Movie 5

Supplemental Movie 6

Supplemental Movie 7

Supplemental Movie 8

Supplemental Movie 9

Supplemental Movie 10

Supplemental Movie 11

Supplemental Movie 12

## Data Availability

The data that support the findings of this study are available from the corresponding author upon reasonable request.
