# Supplementary material for: Self‐Induced Solutal Marangoni Flows Realize Coffee‐Ring‐Less Quantum Dot Microarrays with Extensive Geometric Tunability and Scalability
Source: Adv Sci (Weinh). 2022 Feb 7;9(11):2104519. doi: 10.1002/advs.202104519 (PMC9008421; doi:10.1002/advs.202104519)
Supplement: Supplementary file 1 — Supporting Information [file ADVS-9-2104519-s009.pdf]

ADVANCED SCIENCE
Open Access

# Supporting Information

for *Adv. Sci.*, DOI: 10.1002/advs.202104519

# Self-Induced Solutal Marangoni Flows Realize Coffee-Ring-Less Quantum Dot Microarrays with Extensive Geometric Tunability and Scalability

*Jeongsu Pyeon, Kyeong Min Song, Yeon Sik Jung, and Hyoungsoo Kim**

# Self-induced solutal Marangoni flows realize coffee-ring-less quantum dot microarrays with extensive geometric tunability and scalability: Supplementary information

*Jeongsu Pyeon  Kyeong Min Song  Yeon Sik Jung  Hyoungsoo Kim**

J. Pyeon, H. Kim
Department of Mechanical Engineering, Korea Advanced Institute of Science and Technology, Daejeon 34141, Republic of Korea
Email: hshk@kaist.ac.kr
K. M. Song, Y. S. Jung
Department of Materials Science and Engineering, Korea Advanced Institute of Science and Technology, Daejeon 34141, Republic of Korea

Keywords: *Solutal Marangoni effect, polygonal quantum dot light-emitting diodes, coffee-ring, vertex, vortex pair, confinement effect*

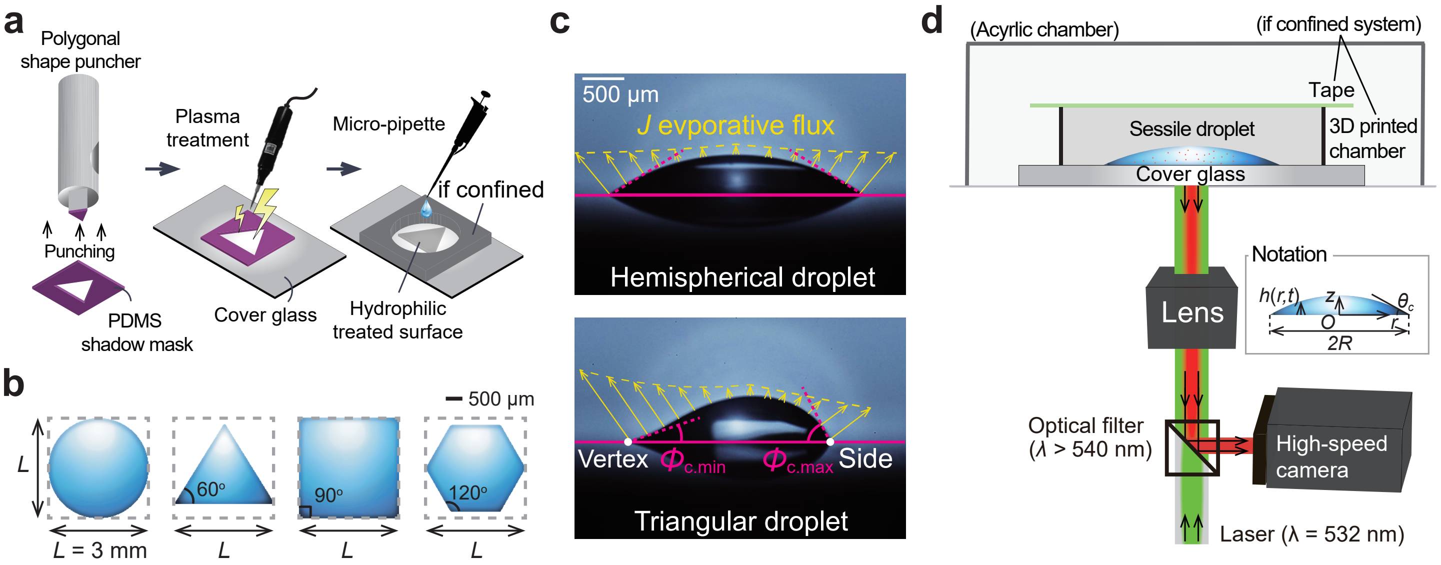


**Figure S1**. Experimental set-up. a) Preparation of polygonal liquid droplets on a cover glass. b) Different shapes of wetting areas where $L$ is the representative length. c) Comparison of evaporative flux distribution and cross-sectional images. d) Experimental set-up for particle image velocimetry (PIV).

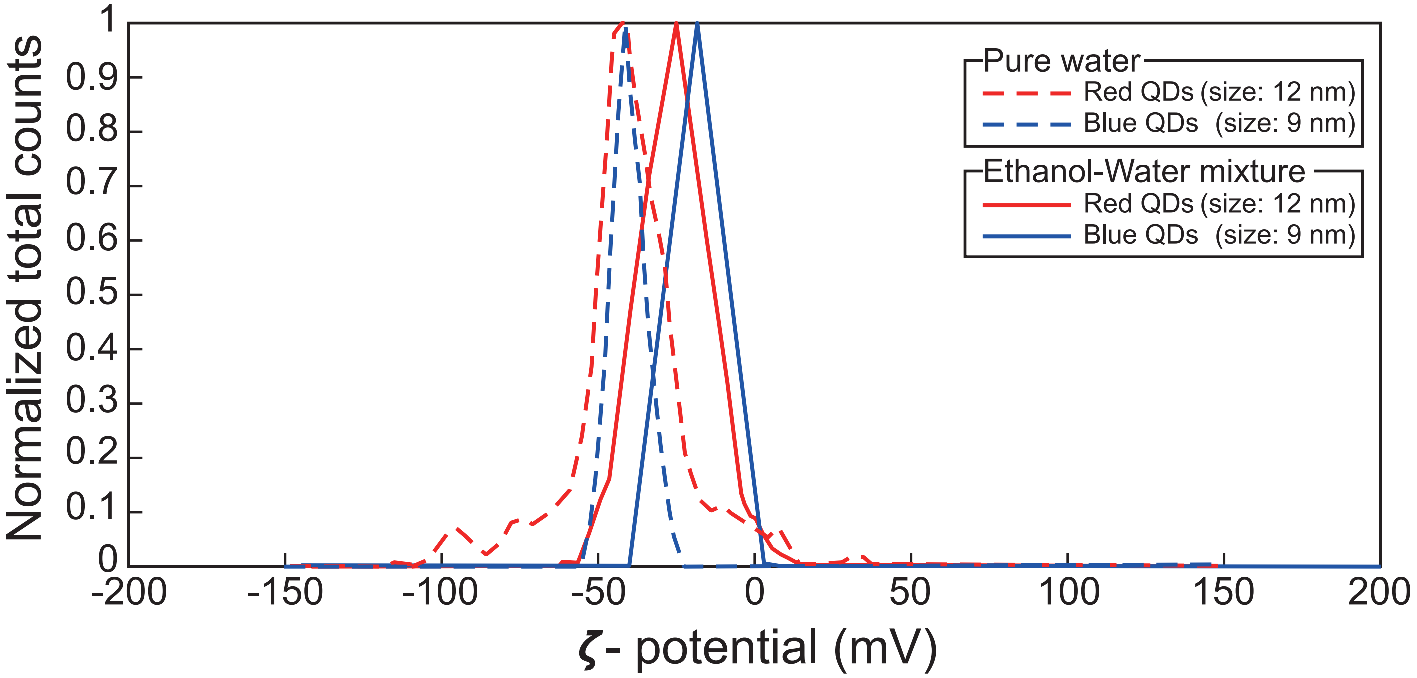


**Figure S2**. Results of $\zeta$-potential measurements of the QD solutions with pure water (dashed lines) or an ethanol-water mixture (solid lines). The mixing ratio of the ethanol, water, and QDs is 48.6 : 51.1 : 0.3 wt%. Here, the total counts $N$ are normalized from 0 to 1 using a following formula: $(N - N_{\mathrm{min}})\,/\,(N_{\mathrm{max}} - N_{\mathrm{min}})$. The $\zeta$-potentials and deviations are red QDs (in pure water): -39.9 mV and 19.4 mV, blue QDs (in pure water): -39.8 mV and 5.58 mV, red QDs (in the binary mixture): -25.4 mV and 10.8 mV, and blue QDs (in the binary mixture): -18.3 mV and 7.43 mV.
